# Supplementary material for: Fostering culture of care for early career researchers—Building a trustful environment: Insights from a German perspective
Source: PLoS One. 2025 Sep 8;20(9):e0331675. doi: 10.1371/journal.pone.0331675 (PMC12416710; doi:10.1371/journal.pone.0331675)
Supplement: Attachment — Submitted filename: reviewer_comments.pdf [file pone.0331675.s003.pdf]

The manuscript deals with the possible health and mental risks for early career researchers (ECRs) who are involved in animal experiments in their research. A brief introduction highlights the role of ECRs, identifies challenges for this group of scientists and speculates on possible causes for these challenges. The experimental part of the study includes a survey with 124 ECRs. The collected responses were analyzed descriptively and provide an overview of the degree of stress, potential causes and applied coping mechanisms. The results are discussed in detail and a number of suggestions for improving the situation are provided.

The present study deals with an important aspect of research that can have a major impact on the individual careers of ECRs, scientific work with animals and the results of such studies. The scientific component of this study may be lower than in other publications, but this is outweighed by its social and scientific relevance. I therefore consider it essential to publish the results of the study.

The article is well written and comprehensible and is particularly convincing due to the thoroughness of the discussion. I only have a few (almost picky) suggestions for changes.

Line 137
12 participants in 3 groups of 3 doesn't add up – please correct numbers

Line 297
This section begins somewhat unfavorably and needs to be formulated clearly: in the methods section participants were described as homogenous group (senior staff, older than 40) different from ECRs however, the question posed in the workshop part 1 suggests the perspective of the ECRs. Perhaps the text should be reworded here so that it is clear who comes to this conclusion.

Line 339
ECS – wrong abbreviation

Line 548
change "labor" to "working" or "laboratory"
